# A tandem motif-based and structural approach can identify hidden functional phosphodiesterases

**DOI:** 10.1016/j.csbj.2021.01.036

**Published:** 2021-01-26

**Authors:** Mateusz Kwiatkowski, Aloysius Wong, Anna Kozakiewicz, Christoph Gehring, Krzysztof Jaworski

**Affiliations:** a Chair of Plant Physiology and Biotechnology, Faculty of Biological and Veterinary Sciences, Nicolaus Copernicus University in Toruń, Lwowska St. 1, 87-100 Toruń, Poland; b Department of Biology, College of Science and Technology, Wenzhou-Kean University, 88 Daxue Road, Ouhai, Wenzhou, Zhejiang Province 325060, China; c Zhejiang Bioinformatics International Science and Technology Cooperation Center of Wenzhou-Kean University, China; d Department of Biomedical and Polymer Chemistry, Faculty of Chemistry, Nicolaus Copernicus University in Toruń, Gagarina St. 7, 87-100 Toruń, Poland; e Department of Chemistry, Biology and Biotechnology, University of Perugia, Borgo XX giugno, 74, 06121 Perugia, Italy

**Keywords:** Phosphodiesterase (PDE), Adenylate cyclase (AC), AtKUP5, AC-PDE activity, Calmodulin (CaM), Moonlighting protein

## Abstract

Cyclic nucleotide monophosphates (cNMPs) are increasingly recognized as essential signaling molecules governing many physiological and developmental processes in prokaryotes and eukaryotes. Degradation of cNMPs is as important as their generation because it offers the capability for transient and dynamic cellular level regulation but unlike their generating enzymes, the degrading enzymes, cyclic nucleotide phosphodiesterases (PDEs) are somewhat elusive in higher plants. Based on sequence analysis and structural properties of canonical PDE catalytic centers, we have developed a consensus sequence search motif and used it to identify candidate PDEs. One of these is an *Arabidopsis thaliana* K^+^-Uptake Permease (AtKUP5). Structural and molecular docking analysis revealed that the identified PDE domain occupies the C-terminal of this protein forming a solvent-exposed distinctive pocket that can spatially accommodate the cyclic adenosine monophosphate (cAMP) substrate and importantly, cAMP assumes a binding pose that is favorable for interactions with the key amino acids in the consensus motif. PDE activity was confirmed by the sensitive liquid chromatography tandem mass spectrometry (LC-MS/MS) method. Notably, this activity was stimulated by the Ca^2+^/CaM complex, the binding of which to the PDE center was confirmed by surface plasmon resonance (SPR). Since AtKUP5 also has adenylate cyclase (AC) activity that is essential for K^+^ transport, we propose that this dual moonlighting AC-PDE architecture, offers modulatory roles that afford intricate intramolecular regulation of cAMP levels thereby enabling fine-tuning of cAMP signaling in K^+^ homeostasis.

## 1 Introduction

In plants, much like in animals, cyclic nucleotide monophosphates (cNMP) and in particular 3′,5′-cyclic adenosine monophosphate (cAMP) and 3′,5′-cyclic guanosine monophosphate (cGMP) are key signaling molecules that trigger many physiological and developmental responses [1], [2] and molecular mechanisms notably cyclic nucleotide monophosphate-dependent phosphorylation [3], [4], [5]. Despite the growing evidence for the role of cAMP in many plant physiological processes, the molecular mechanism of cAMP metabolism remains poorly understood.

In the animal cell, cNMP homeostasis is achieved by the coordinated action of cyclases responsible for the synthesis and cyclic nucleotide phosphodiesterases (cNMP PDEs), enzymes capable of degrading 3′,5′-cNMPs to inactive 5ʹ- and 3ʹ-NMPs [6]. Cyclic NMPs regulate processes by binding to and activating cNMP effectors, such as cAMP- and cGMP-dependent protein kinases (PKA and PKG) [7] or bind to cyclic nucleotide-gated channels (CNGCs) which are responsible for conducting Ca^2+^ ions in signal transduction [8].

In higher plants, while the cAMP generating enzymes (adenylate cyclases (ACs)) have been identified in recent years, the degrading enzymes (the PDEs) have remained somewhat elusive. To-date, only one PDE which is a cGMP-dependent AtCN-PDE1 (At1g17330), was reported in higher plants. This PDE, which belongs to the HD-domain/PDEase-like protein superfamily with orthologs in bacteria and archaea, responds to UV and is implicated in stomatal movement [9], [10].

Here we propose that there are more PDEs in the *A. thaliana* proteome that remain to be discovered and through a combination of complementary computational and experimental approaches, we present an amino acid consensus search motif that can, in tandem with structural and molecular docking assessments, identify seemingly hidden catalytically active PDE centers in complex proteins.

## 2 Results and discussion

### 2.1 Identification of candidate PDEs in *A. thaliana*

**Fig. 1:**
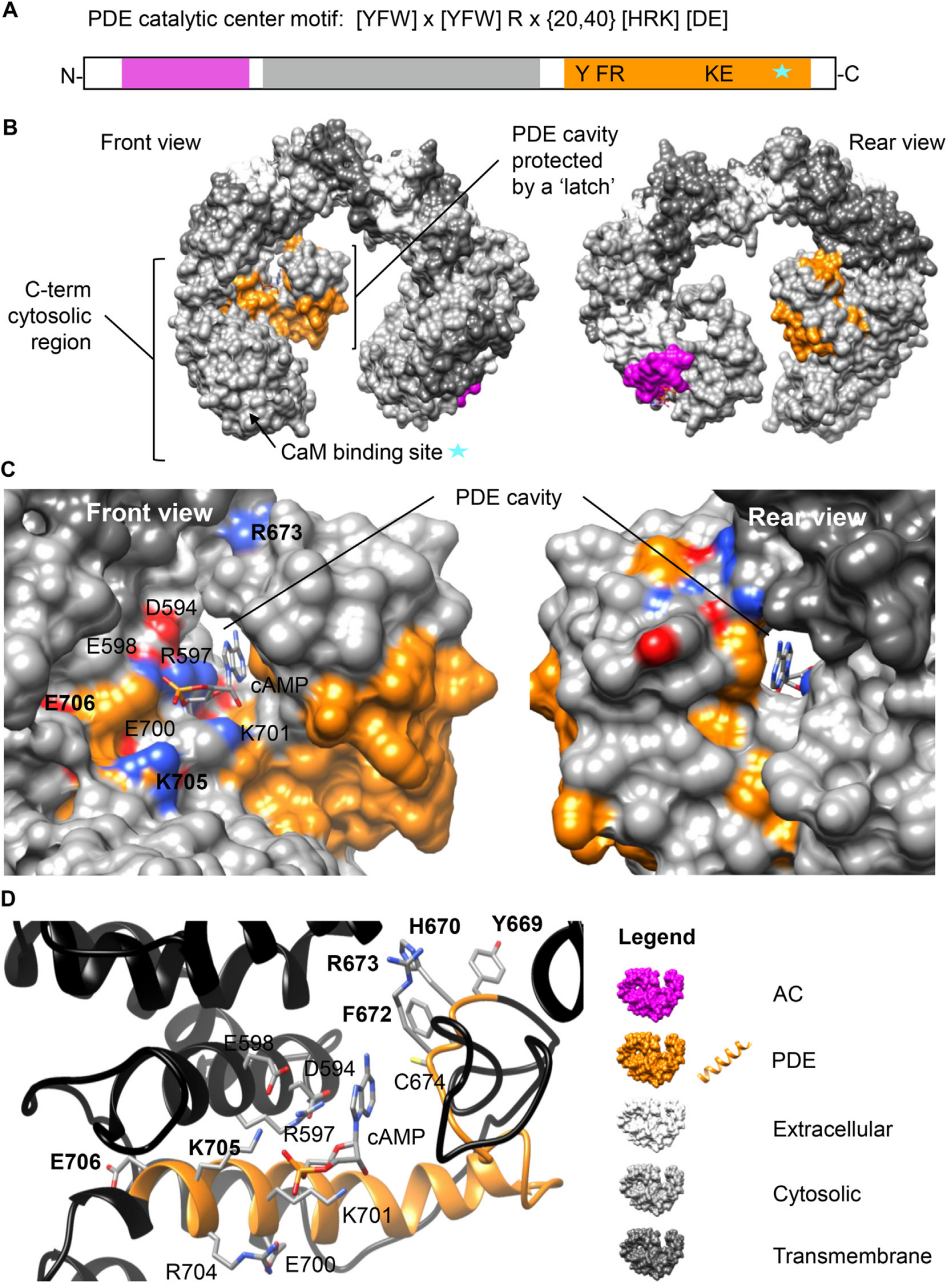
Computational assessment of the PDE center in AtKUP5. (A) The amino acid motif of annotated and experimentally tested PDE catalytic centers. The following box diagram presents the amino acid sequence of the AtKUP5 K^+^ transporter encoding a PDE and AC. The PDE domain is located in the cytosolic C terminal region and is shown in orange, with critical residues of the motif and calmodulin binding domain (blue star). The AC catalytic center is located in the cytosolic N terminal region and is shown in purple. The 12 transmembrane domains are shown in gray. (B) The full-length AtKUP5 model showing the location of the PDE domain at the solvent-exposed cytosolic C-terminal region. Dockings and interactions of cAMP with key residues at the PDE domain of AtKUP5 are shown as (C) surface and as (D) ribbon models respectively. The PDE domain is highlighted in orange and the amino acid residues implicated in interactions with cAMP are colored according to their charges in the surface models and shown as individual atoms in the ribbon model. Key amino acids in the PDE motif [YFW]Hx[YFW]Rx{20,40}[HRK][DE] are bolded and other amino acids in the vicinity capable for interaction with cAMP are labeled accordingly. The full-length AtKUP5 structure was modeled against the AtKUP7 template [28] using the MODELLER (ver. 9.25) software [29]. cAMP docking simulations were performed using AutoDock Vina (ver. 1.1.2) [30]. Molecular graphics and analyses were performed with the UCSF Chimera package [31]. Orientations and substrate binding poses were analyzed with the UCSF Chimera package [31]. Chimera was developed by the Resource for Biocomputing, Visualization, and Informatics at the University of California, San Francisco (supported by NIGMS P41-GM103311). (For interpretation of the references to colour in this figure legend, the reader is referred to the web version of this article.)

We hypothesized that catalytically active PDE centers in *A. thaliana* exist in complex multifunctional proteins masked by much larger primary domains and are thus undetected by homology searches. In order to detect them, we aligned PDE catalytic centers from annotated PDEs from yeast and animals and extracted a consensus search term (motif) (Fig. 1A). The motif [YFW]Hx[YFW]Rx{20,40}[HRK][DE] was then used to query the *A. thaliana* proteome and retrieved 32 candidates not including splice variants (Supplementary Table S1). This type of motif-based search method has previously identified functional centers of diverse molecular functions in complex proteins [11], [12], [13]. Additional details and specific steps (and alignments) for the identification of candidate PDEs are provided in Supplementary Methods. Within these candidates we noted two K^+^ transporters which also have AC activities and chose AtKUP5 [14] for further investigation.

### 2.2 Computational evaluation of a candidate PDE

AtKUP5 contains an AC domain located in the N-terminal cytosolic domain with the catalytic core spanning amino acids from 81 to 96 (Fig. 1A) while the PDE catalytic center is located in the C-terminus like calmodulin binding (amino acids 814 – 834) domain. To probe the PDE center, we used the full length AtKUP5 model for structural assessment and cAMP substrate docking simulations. The motif [YFW]Hx[YFW]Rx{20,40}[HRK][DE] appears at the C-terminal (Y669 – E706) in the cytosolic region, and assumes a “helix-loop” secondary fold (Fig. 1). At the tertiary level, the PDE domain contains a cavity protected by a ‘latch’ (Fig. 1B). Importantly, cAMP docks at the domain with mean binding affinity of −6.02 ± 0.02 kcal/mol and can assume a binding pose that is spatially favorable for interactions with key amino acids in the motif. We consider a “correct binding pose” an orientation where the adenine head of cAMP points towards [YFW]Hx[FWY]R and the phosphate tail points towards [HRK] of the PDE motif. This rationale was based on the functional roles of corresponding amino acids in annotated plant PDEs i.e., *Physcomitrella patens*, *Marchantia polymorpha* and *Arabidopsis thaliana*, from which the PDE motif was constructed (see Supplementary Methods). In AtKUP5, the phosphate of cAMP points towards K705 and the adenine points towards H670 (Fig. 1C and D) and notably, this binding pose has a 72.2% docking frequency in AtKUP5 (Supplementary Figure S2). Key amino acids in the motif, Y669, H670, F672, R673, K705 and E706 are bolded and, other amino acids in the vicinity deemed capable for interactions with cAMP are labeled accordingly (Fig. 1C and D).

We also assess a close homolog of AtKUP5, AtKUP7, that also harbors a PDE center. AtKUP5 shares 73.8% identity with AtKUP7 and assumes a similar structural fold with AtKUP5 at the PDE center. The PDE center is located at the C-terminal (Y668 – E705) in the cytosolic region and assumes a “helix-loop” secondary fold, while at the tertiary level, the PDE domain contains a cavity protected by a ‘latch’ much like in AtKUP5. Importantly, cAMP docks at the domain with a mean binding affinity of −6.72 ± 0.01 kcal/mol and assumes a positive binding pose frequency of 94.4%. The key amino acids in the motif, Y668, H669, F671, R672 and E705 also appear in AtKUP7 with the exception that K705 in AtKUP5 is replaced by R704 in AtKUP7 (Supplementary Figure S2).

Further, we examined two Arabidopsis proteins AtLARP1 (At5g21160) and AtKING1 (At3g48530) which were among seven PDE candidates identified by the PDE motif [YFW]Hx[YFW]Rx{20,40}[HRK][DE] (Supplementary Methods). Docking data and representative structures of AtLARP1A and AtKING1 PDE centers docked with cAMP are presented in Supplementary Figure S3. Unlike AtKUP5 and AtKUP7, these two PDE candidates do not contain a “cleft-latch” like structure but the PDE motif occupies clear cavities that spatially accommodate cAMP with mean binding affinities of −4.60 ± 0.06 kcal/mol for AtLARP1A and −5.14 ± 0.34 kcal/mol for AtKING1, respectively. Structural assessments also indicate that the gap x{20,40} between [YFW]Hx[YFW]R and [HRK] in the PDE motif, might be narrower in AtLARP1A and AtKING1. For instance, in AtLARP1A, the phosphate end of cAMP appears to be interacting with K802 which is only 17 amino acids apart while in AtKING1, this gap is only 12 (Supplementary Figure S3). Future experimental validations of candidates with different structural folds than AtKUP5 will guide refinement of the existing PDE motif.

Computational analysis suggests that the PDE motif appears in structurally diverse proteins with varying primary functions e.g., K^+^ transport, RNA-binding and kinase; however, they could accommodate cAMP substrate with good binding affinities (mean free energies ranging from −4.60 kcal/mol for AtLARP1A to −6.72 ± 0.01 kcal/mol for AtKUP7) within clear cavities. We propose that the PDE motif identifies catalytic centers that operate as modulators at moonlighting sites in their respective proteins of varying primary functions [15] and it is therefore conceivable that they assume diverse structural folds which are different than the existing classes of PDEs. We note that since the strength of this motif-based approach is to enable the identification of PDE centers hidden within complex proteins which would otherwise be undiscovered through standard homology approaches, experimental validations will be required to ascertain the functionality, substrate selectivity and catalytic rates of candidates identified through this approach.

### 2.3 Biochemical evaluation of PDE activity in AtKUP5

**Fig. 2:**
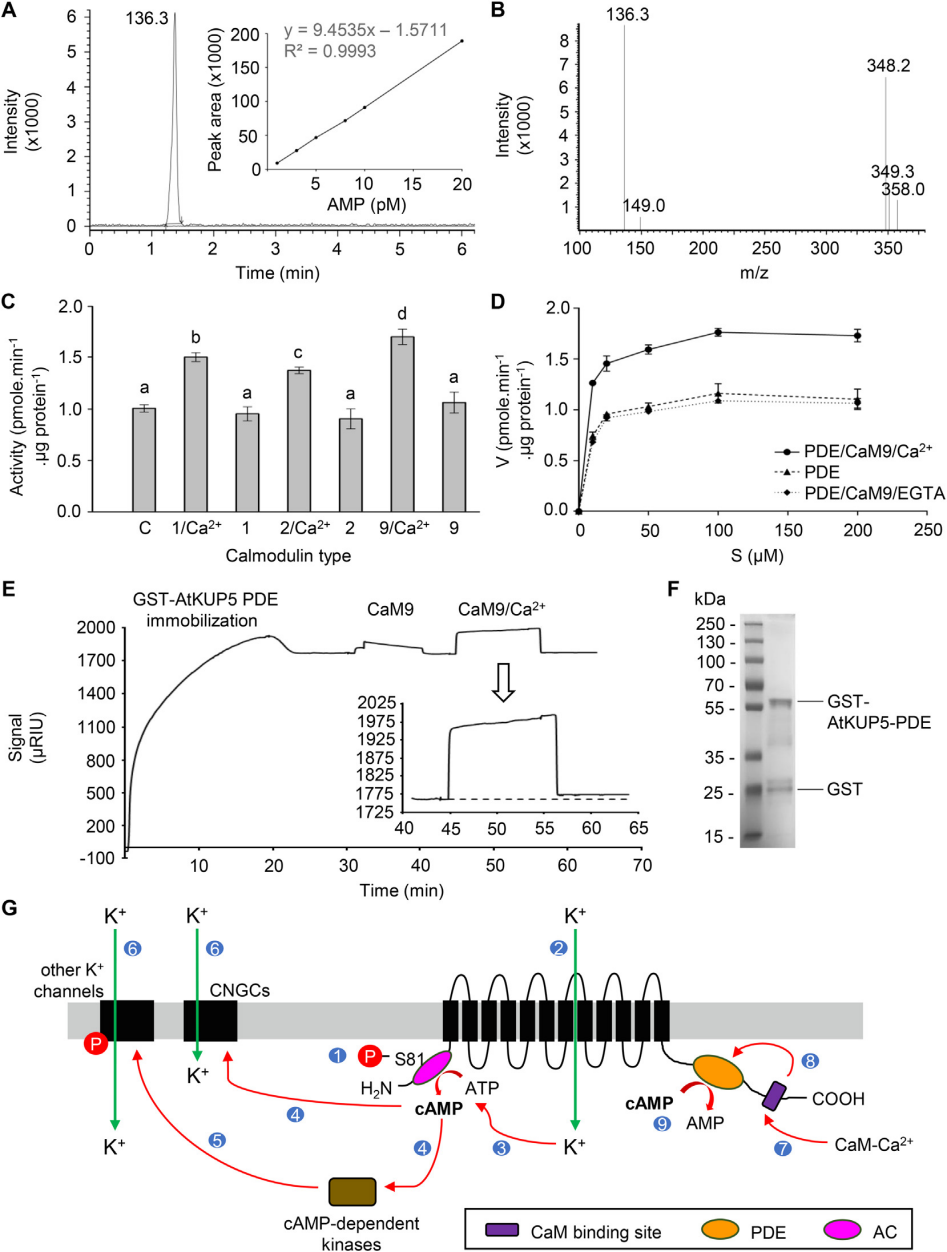
PDE activity of AtKUP5 and a model for the dampening role of PDE in K^+^ homeostasis. (A) Ion chromatogram of AMP together with calibration curve. (B) Inset showing the parent AMP ion at *m*/*z* 348.20 [M + H]^+^ and the corresponding fragmented daughter ion at *m*/*z* 136.30 [M + H]^+^. The fragmented product ion was used for quantitation. (C) GST-AtKUP5^573-855^ activity in the presence of various CaM isoforms, with or without addition of Ca^2+^ ions. Data are mean values (n = 3) and error bars show standard error of the mean. Statistical analysis was performed by one-way ANOVA followed by a Tukey–Kramer multiple comparison test. Different letters indicate significantly different data. (D) Michaelis-Menten plot of the phosphodiesterase activity of GST-AtKUP5^573-855^, in the presence of CaM9/Ca^2+^ and CaM9/Ca^2+^ with addition of an ion chelating compound EGTA, that is intended to prevent the formation of the CaM active complex. Values indicate means ± SD (n = 3). (E) Binding analysis of CaM9 to GST-AtKUP5^573-855^ on the surface of gold discs covered with glutathione. The increasing μRIUs reflect the mass change on the surface of modified gold disc, i.e., the binding of the GST-AtKUP5^573-855^, CaM9, and CaM9/Ca^2+^ complex, respectively. The signal rapidly increases and then plateaus as the system reaches equilibrium. When the injection ends, the CaM9 and CaM9/Ca^2+^ complex dissociates, resulting in a decrease in the signal. (F) The purified GST-AtKUP5^573-855^ protein (3 μg) was analyzed by SDS-PAGE. (G) A model for the dampening role of PDE in the regulation of K^+^ transport. It was previously speculated that phosphorylation of S81 (1), Y92, T75 and/or S76 located within or close to the AC center of AtKUP5, activates its K *+* transport activity (2). K^+^ uptake will in turn stimulate the AC activity of AtKUP5, converting ATP into the second messenger cAMP [14] (3). cAMP will orchestrate a cascade of cellular signaling pathways through the activation of cAMP-dependent protein kinases (4) which can phosphorylate and thus activating, other K^+^ channels (5), thereby further increasing cytosolic K^+^ (6). Alternatively, cAMP can also activate cyclic nucleotide gated channels (CNGCs) (4) to directly increase the transport of K^+^ into the cell (6). Ca^2+^ activated calmodulin (CaM-Ca^2+^) can bind to the C-terminal of AtKUP5 (7) and activates the activity of the PDE (8) (shown in this study), converting cAMP into AMP. Degradation of cAMP by PDE dampens the cAMP-mediated signaling processes (9) that include K^+^ transport.

PDE catalytic activity was assessed by enzymatic assays with cAMP as the substrate and the AMP product was detected and quantified using the sensitive liquid chromatography tandem mass spectrometry (LC-MS/MS) method (Fig. 2A). AtKUP5^573-855^ generates AMP from the substrate cAMP with a V_max_ of 1.17 pmole AMP min^−1^ μg protein^−1^ (0.25 mmol AMP s^−1^ mol protein^-1^), and a K_m_ of 5.35 µM. Cyclic GMP is not a substrate for AtKUP5^573-855^ (Supplementary Figure S1). Comparing the obtained results with other plant PDEs, we noted that AtKUP5 has the lowest rate e.g., the PDE of MpCAPE has 100x higher activity [16] while the V_max_ of AtCN-PDE1 is 50x greater [9].

We also assessed the effect of Ca^2+^ and CaM isoforms derived from *A. thaliana* and bovine brain on the PDE activity. All CaM isoforms stimulate cAMP hydrolysis, after the formation of the active CaM/Ca^2+^ complex (Fig. 2C) where four calmodulin EF-hand motifs bind single Ca^2+^ ion [17]. The addition of the active CaM/Ca^2+^ complex causes increases in the V_max_ (1.78 pmole min^−1^ μg protein^−1^) and the affinity of the enzyme for the substrate (K_m_ 4.24 µM) (Fig. 2D). The formation of the active CaM/Ca^2+^ complex is not possible when using EGTA, which chelates Ca^2+^ ions. This results in no noticeable increase in PDE activity despite the addition of CaM/Ca^2+^.We then used surface plasmon resonance (SPR) to assess the binding affinity of CaM to PDE. Fig. 2E shows the sensogram for interactions between immobilized GST-AtKUP5^573-855^ and CaM9 without, and in the presence of, Ca^2+^ ions. In both cases, an increase in signal was observed, but for the binding of CaM/Ca^2+^ complex the increasing µRIUs was higher and signal amplitude did not decline to the baseline level after sample washing (11 µRIU difference). This implies that the CaM/Ca^2+^ complex binds to AtKUP5^573-855^.

### 2.4 Conclusion and outlook

Considering our current findings in the context of the whole protein and its broader cellular signaling functions, we propose a model (Fig. 2G) where the PDE has a dampening role on AtKUP5 in K^+^ homeostasis. It was previously speculated that phosphorylation of S81, Y92, T75 and/or S76 located within or close to the AC center of AtKUP5, activates its K^+^ transport activity [18], [19], [20], [21]. K^+^ uptake will in turn stimulate the AC activity of AtKUP5, converting ATP into the second messenger cAMP [14]. Cyclic AMP will orchestrate a cascade of cellular signaling pathways either, 1) through the activation of cAMP-dependent protein kinases which can phosphorylate and thus activate other K^+^ channels thereby further increasing cytosolic K^+^ [22] or, 2) by activating cyclic nucleotide gated channels (CNGCs) to directly increase the transport of K^+^ into the cell [23], [24], [25]. Ca^2+^ activated calmodulin (CaM-Ca^2+^) is predicted to bind at the C-terminal of AtKUP5 which in turn activates the PDE (Fig. 2C-E), thus affecting K^+^ net flux and homeostasis (Fig. 2G).

In conclusion, we have constructed a consensus search motif for the identification of candidate PDEs based on key amino acids directly involved in the molecular function of ligand binding sites that are conserved in various species. This method has identified novel PDE candidates in *A. thaliana*. It appears that many PDEs, much like ACs, are part of multifunctional multi-domain proteins. One of the candidates is AtKUP5 which has been previously shown to have AC activity essential for its role as a K^+^ transporter. Incidentally, a similar domain combination, AC-PDE, was recently reported in the liverwort *Marchantia polymorpha* [16]. It is thus conceivable that this domain architecture represents an ancient signaling module where one protein can transiently and dynamically regulate localized cAMP concentration. Taken together, our results have assigned the PDE center in AtKUP5 to a dampening role of the cAMP signal and we propose that this dual AC-PDE architecture, affords intricate control and concomitantly also the fine-tuning of cellular cAMP signaling and in the broader K^+^ homeostasis.

## 3 Material and methods

### 3.1 Construction of a PDE consensus sequence motif

For the identification of *A. thaliana* candidate PDEs we used a method based on motif searches as described previously [26], [27]. The motif was built from an alignment of key residues in the catalytic center of annotated PDEs from different species. The motif ([YFW]Hx[YFW]Rx{20,40}[HRK][DE]) was used to query Swiss-Prot and PatMatch function in The Arabidopsis Information Resource (TAIR). Amino acids between square brackets are those that are allowed in a position, “x” stands for any amino acid and curly brackets delineate the number of undetermined amino acids. Detailed description and steps for the construction of the PDE motif are provided in Supplementary Methods. The calmodulin binding site was predicted using Calmodulin Target Database at Cellular Calcium Information Server (http://calcium.uhnres.utoronto.ca/).

### 3.2 Structural analysis of the PDE center in AtKUP5

The full-length AtKUP5 structure was modeled against the AtKUP7 template as described in [28] using the MODELLER (ver. 9.25) software [14], [28], [29]. Docking simulations of cAMP to the PDE domain of AtKUP5 was performed using AutoDock Vina (ver. 1.1.2) [30]. In docking simulations, all bonds in the cAMP ligand were allowed to move freely but the protein was set rigid. Docking orientations of cAMP were evaluated based on a pre-determined “correct binding pose” where the orientation of cAMP deemed favorable for catalysis is presumably to be as follows: adenine head pointing towards [YFW]Hx[FWY]R and the phosphate tail pointing towards [HRK] of the PDE motif. This rationale was based on the functional roles of corresponding amino acids in annotated plant PDEs i.e., *Physcomitrella patens*, *Marchantia polymorpha* and *Arabidopsis thaliana*, from which the PDE motif was constructed (Supplementary Methods). Docking simulations consider both spatial and charge at the vicinity of the catalytic center based on pre-determined grids that cover the entire PDE center and can afford free rotation of cAMP substrate which we have been set prior to docking experiments. The cAMP docking poses were analyzed, and all images created by UCSF Chimera (ver. 1.10.1) [31]. Chimera was developed by the Resource for Biocomputing, Visualization, and Informatics at the University of California, San Francisco (supported by NIGMS P41-GM103311). All 3D structures and the PDE domains of AtKUP7, AtLARP1A and AtKING1 were generated in the same manner by homology modeling using the MODELLER (ver. 9.25) software [29] and docked to cAMP using AutoDock Vina (ver. 1.1.2) [30]. All structures, binding poses, and images were analyzed and created using UCSF Chimera (ver. 1.10.1) [31].

### 3.3 PDE biochemical assay and LC-MS/MS detection

PDE *in vitro* activity was determined by using LC-MS/MS to determine the rate of AMP formation. The reaction mixture contained: 3 mM Tris-HCl (pH 8.0), 0,1 mM cAMP, 0.1% (v/v) 2-mercaptoethanol, 5 µg of GST-AtKUP5^573-855^, 0.5 mM MgCl_2_ and MnCl_2_. To check if calmodulin regulates the PDE activity, three different CaM isoforms (*A. thaliana* CaM1, CaM9 and bovine CaM2) were added to the reaction in the concentration of 2 µM and the GST-AtKUP5^573-855^ protein concentration was 0,625 µM. Samples were incubated at 37 °C for 25 min. The enzyme reaction was terminated by incubation at 100 °C for 10 min and the samples were centrifuged at 13,200 × g for 10 min.

LC-MS/MS experiments were performed using the Nexera UHPLC and LCMS-8045 integrated system (Shimadzu Corporation). The ionization source parameters were optimized in positive ESI mode using pure AMP dissolved in HPLC-grade water (Sigma). Samples were separated using a ReproSil Star 100 ZIK HiliC column (150 × 2 mm, 3 µm, Dr. Maisch GmbH). A gradient of solvent A (0.1% (v/v) formic acid with 25 mM ammonium formate) and solvent B (100% (v/v) acetonitryle) was applied over 6 min: B: 95% − 35% with a flow rate of 0.6 mL/min. The interface voltage was set at 4.0 kV for positive (ES + ) electrospray. Data acquisition and analysis were done with LabSolutions workstation for LCMS-8045.

## Funding

M.K. was supported from the project POWR.03.05.00–00-Z302/17 Universitas Copernicana Thoruniensis in Futuro-IDS “Academia Copernicana”. A.W. was supported by the National Natural Science Foundation of China (31850410470) and the Zhejiang Provincial Natural Science Foundation of China (LQ19C130001).

## CRediT authorship contribution statement

**Mateusz Kwiatkowski:** Conceptualization, Methodology, Investigation, Writing - original draft, Funding acquisition. **Aloysius Wong:** Conceptualization, Formal analysis, Visualization, Funding acquisition, Writing - original draft, Writing - review & editing. **Anna Kozakiewicz:** Methodology, Investigation. **Christoph Gehring:** Conceptualization, Writing - original draft, Writing - review & editing, Supervision. **Krzysztof Jaworski:** Conceptualization, Writing - original draft, Writing - review & editing, Supervision.

## Declaration of Competing Interest

The authors declare that they have no known competing financial interests or personal relationships that could have appeared to influence the work reported in this paper.
