# Supplementary material for: A tandem motif-based and structural approach can identify hidden functional phosphodiesterases
Source: Comput Struct Biotechnol J. 2021 Jan 26;19:970–5. doi: 10.1016/j.csbj.2021.01.036 (PMC7873575; doi:10.1016/j.csbj.2021.01.036)
Supplement: Supplementary data 1 [file mmc1.docx]

**Supplementary Information**

**A tandem motif-based and structural approach can identify hidden functional phosphodiesterases**

Mateusz Kwiatkowski^a^, Aloysius Wong^b, c^, Anna Kozakiewicz^d^, Christoph Gehring^e^, Krzysztof Jaworski^a,^ *

^a^ Chair of Plant Physiology and Biotechnology, Nicolaus Copernicus University, Lwowska St. 1, 87-100 Torun, Poland

^b^ Department of Biology, College of Science and Technology, Wenzhou-Kean University, 88 Daxue Road, Ouhai, Wenzhou, Zhejiang Province 325060, China

^c^ Zhejiang Bioinformatics International Science and Technology Cooperation Center of Wenzhou-Kean University, China

^d^ Department of Biomedical and Polymer Chemistry, Faculty of Chemistry, Nicolaus Copernicus University, Gagarina St. 7, 87-100 Torun, Poland

^e^ Department of Chemistry, Biology and Biotechnology, University of Perugia, Borgo XX giugno, 74, 06121 Perugia, Italy

*Corresponding author: Tel: +48-56-611-44-56; Fax: +48-56-61-14-772; E-mail: [jaworski@umk.pl](mailto:jaworski@umk.pl)

**Supplementary Information contains three figures, one table, supplementary methods and supplementary references**


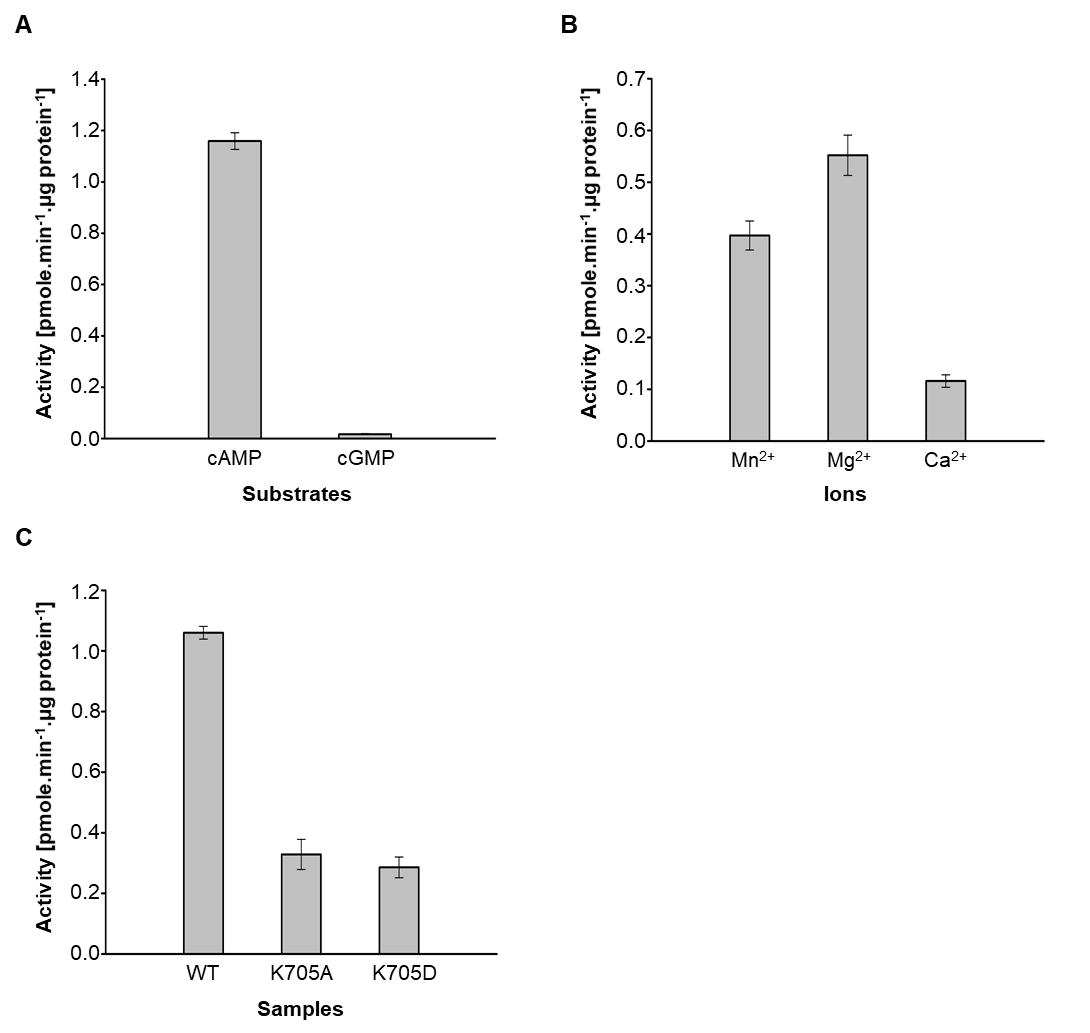
**Supplementary Figure S1**

**PDE activities of AtKUP5. (A)** Substrate specificity of GST-AtKUP5 PDE. Phosphodiesterase activity measured after 30 minutes at 37 ^o^C in the presence of 100 µM cAMP or 100 µM cGMP. Data are mean values (n = 3) and error bars show standard error of the mean. **(B)** Effect of divalent cations on phosphodiesterase activity of GST-AtKUP5 PDE. PDE activity of GST-AtKUP5 protein was measured in the presence of various cations, where cAMP was used as substrate. Data represent means ± SD (n = 3). **(C)** Effect of lysine (K) 705 mutation on PDE activity. Mutations of the lysine to alanine (A) and aspartate (D) in the wild-type (WT) AtKUP5 PDE domain significantly reduced enzyme activity.

**Supplementary Figure S2**

| **AtKUP5 cAMP docking solutions** | | | | | | | |
| --- | --- | --- | --- | --- | --- | --- | --- |
| Mode  1^st^ run | Affinity | Distance from best mode | | Mode  2^nd^ run | Affinity | Distance from best mode | |
|  | (kcal/mol) | rmsd l.b. | rmsd u.b. |  | (kcal/mol) | rmsd l.b. | rmsd u.b. |
| 1 | -7.7 | 0.000 | 0.000 **╳** | 1 | -7.6 | 0.000 | 0.000 **╳** |
| 2 | -7.2 | 2.010 | 3.487 **╳** | 2 | -7.2 | 2.028 | 3.508 **╳** |
| 3 | -6.6 | 4.434 | 7.083 **✓** | 3 | -6.6 | 4.453 | 7.087 **✓** |
| 4 | -6.1 | 5.951 | 8.659 **✓** | 4 | -6.1 | 4.429 | 7.387 **✓** |
| 5 | -6.0 | 5.934 | 8.420 **✓** | 5 | -6.1 | 5.912 | 8.650 **✓** |
| 6 | -5.9 | 5.947 | 8.641 **✓** | 6 | -6.1 | 5.823 | 8.298 **✓** |
| 7 | -5.8 | 5.465 | 7.572 **✓** | 7 | -5.8 | 6.599 | 9.326 **✓** |
| 8 | -5.8 | 5.275 | 7.795 **✓** | 8 | -5.7 | 5.675 | 8.411 **✓** |
| 9 | -5.8 | 3.429 | 5.051 **╳** | 9 | -5.6 | 4.858 | 7.814 **✓** |
| Ave ± SD | -6.03 ± 0.30 | | | Ave ± SD | -6.00 ± 0.34 | | |
| **Mean ± SEM** | **-6.02 ± 0.02** | | | | | | |

- solutions with “correct binding pose” where adenine points towards Y669 and phosphate towards R705

**╳** solutions with “incorrect binding pose”

Only solutions with “correct binding pose” are considered in free energy calculations

**Total “correct binding pose” (✓) = 13/18 = 72.2 %**

**Correct cAMP binding pose (13/18 ✓) Incorrect cAMP binding pose (5/18 ╳)**

**1^st^ run**

**
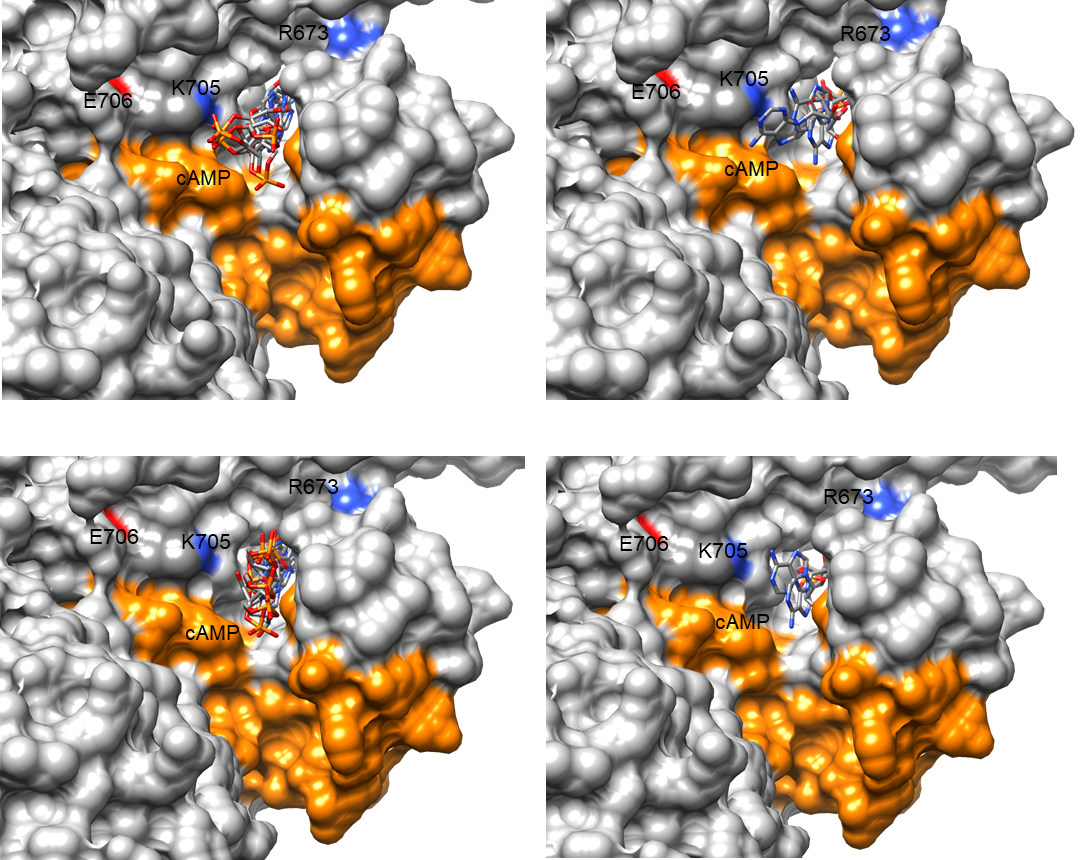

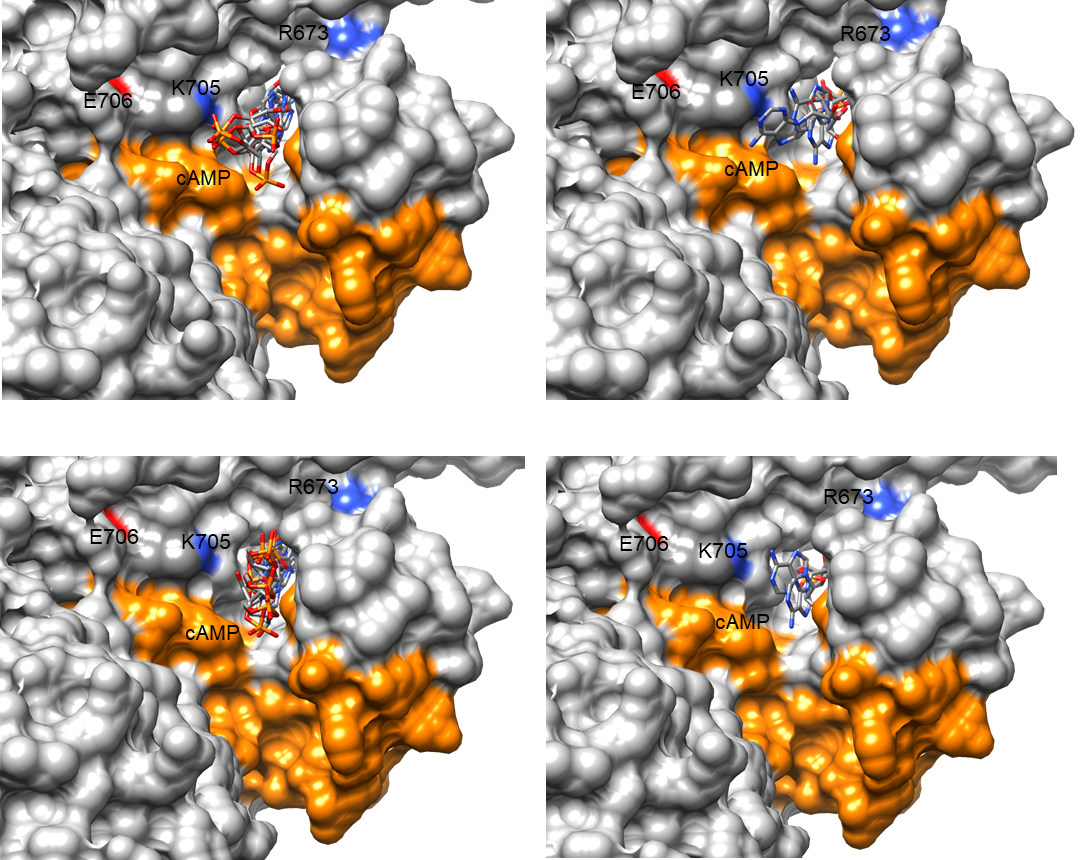
**

**2^nd^ run**

| **AtKUP7 cAMP docking solutions** | | | | | | | |
| --- | --- | --- | --- | --- | --- | --- | --- |
| Mode  1^st^ run | Affinity | Distance from best mode | | Mode  2^nd^ run | Affinity | Distance from best mode | |
|  | (kcal/mol) | rmsd l.b. | rmsd u.b. |  | (kcal/mol) | rmsd l.b. | rmsd u.b. |
| 1 | -7.4 | 0.000 | 0.000 **✓** | 1 | -7.3 | 0.000 | 0.000 **✓** |
| 2 | -7.1 | 2.704 | 3.660 **✓** | 2 | -7.1 | 2.607 | 3.527 **✓** |
| 3 | -6.9 | 1.617 | 1.964 **✓** | 3 | -6.9 | 3.966 | 5.867 **✓** |
| 4 | -6.9 | 4.015 | 5.980 **✓** | 4 | -6.7 | 2.603 | 4.076 **✓** |
| 5 | -6.6 | 3.825 | 5.892 **✓** | 5 | -6.6 | 3.830 | 5.903 **✓** |
| 6 | -6.5 | 4.314 | 5.926 **✓** | 6 | -6.6 | 14.756 | 16.296 **╳** |
| 7 | -6.4 | 4.314 | 5.926 **✓** | 7 | -6.4 | 4.660 | 6.863 **✓** |
| 8 | -6.4 | 5.283 | 6.978 **✓** | 8 | -6.4 | 5.199 | 6.871 **✓** |
| 9 | -6.4 | 2.118 | 3.632 **✓** | 9 | -6.3 | 3.963 | 5.779 **✓** |
| Ave ± SD | -6.73 ± 0.36 | | | Ave ± SD | -6.71 ± 0.36 | | |
| **Mean ± SEM** | **-6.72 ± 0.01** | | | | | | |

- solutions with “correct binding pose” where adenine points towards Y668 and phosphate towards R704

**╳** solutions with “incorrect binding pose”

Only solutions with “correct binding pose” are considered in free energy calculations

**Total “correct binding pose” (✓) = 17/18 = 94.4 %**

**Correct cAMP binding pose (17/18 ✓)**

**1^st^ run**

**
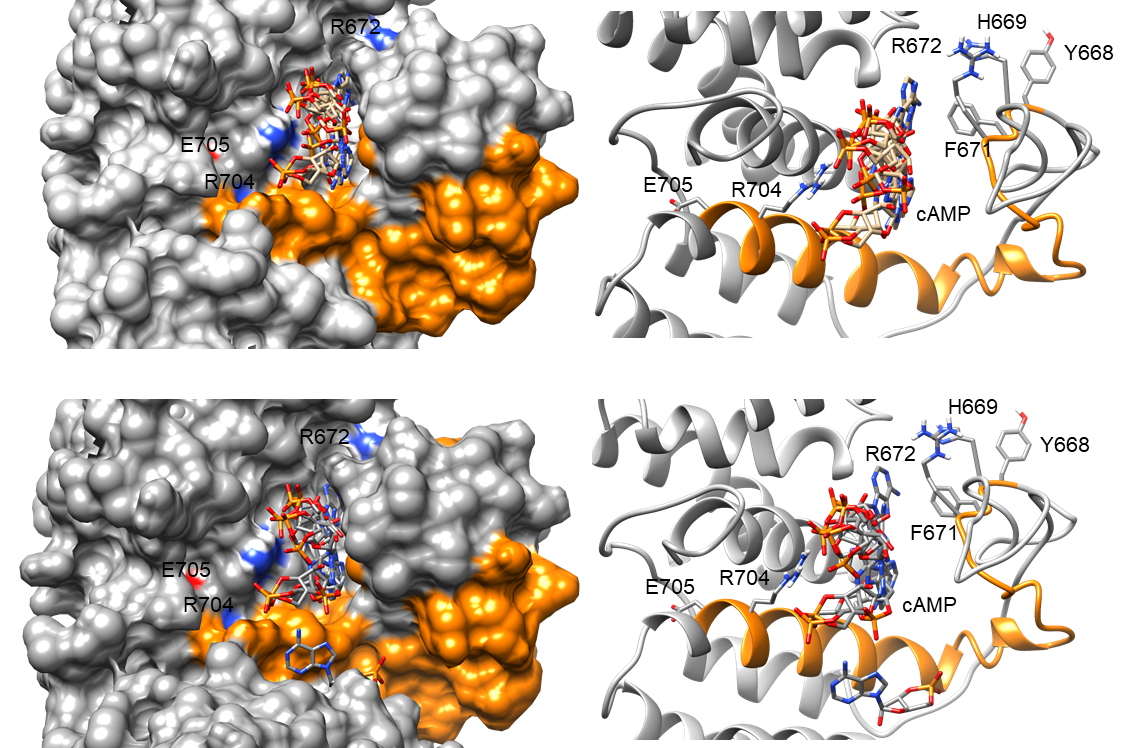
**

**2^nd^ run**

**╳** (incorrect)

AtKUP5 *Arabidopsis thaliana* potassium transporter 13 (TAIR ID: At4g33530; UniProt ID: Q8LPL8)

AtKUP7 *Arabidopsis thaliana* potassium transporter 7 (TAIR ID: At5g09400; UniProt ID: Q9FY75)

**Clusters, docking data of AtKUP5 and AtKUP7, and interpretation of the docking solutions.** Since AtKUP5 shares 73.8% identity with AtKUP7, the full-length AtKUP5 structure was modeled against the AtKUP7 template that has been previously generated in [1] using the MODELLER (ver. 9.25) software [1-3]. AtKUP5 and AtKUP7 were docked with cAMP at their PDE centers. A total of 18 solutions across two independent simulations generated by AutoDock Vina (ver. 1.1.2) [4] were evaluated in terms of free energies and binding poses. Orientations and binding poses were analyzed with the UCSF Chimera (ver. 1.10.1) [5]. Chimera is developed by the Resource for Biocomputing, Visualization, and Informatics at the University of California, San Francisco (supported by NIGMS P41-GM103311). In docking simulations, all bonds in the cAMP ligand were allowed to move freely but the protein was set rigid. Docking orientations of cAMP were evaluated based on a pre-determined "correct binding pose" where the orientation of cAMP deemed favorable for catalysis is presumably to be as follows: adenine head pointing towards [YFW]Hx[FWY]R that is the beginning of the PDE motif and the phosphate tail pointing towards the positively charged residue [HRK] at the end of the motif. This rationale was based on the functional roles of corresponding amino acids in annotated plant PDEs i.e., *Physcomitrella patens*, *Marchantia polymorpha* and *Arabidopsis thaliana*, from which the PDE motif was constructed. Docking simulations consider both spatial and charge at the vicinity of the catalytic center based on pre-determined grids that cover the entire PDE center and can afford free rotation of cAMP substrate which we have set prior to docking experiments. Although varying binding poses of cAMPs may have good binding affinities, not all docking solutions possess the "correct binding pose" that we have ascertained. Thus, we manually analyzed the binding pose of each solution and mark those with the correct binding poses as **✓** and those with incorrect binding poses as **╳** . We also consider how frequent the software finds the “correct binding pose” and found that it is 72.2% for AtKUP5 and 94.4% for AtKUP7 while the mean binding affinities for AtKUP5 and AtKUP7 were -6.02 ± 0.02 and -6.72 ± 0.01 kcal/mol, respectively.

**
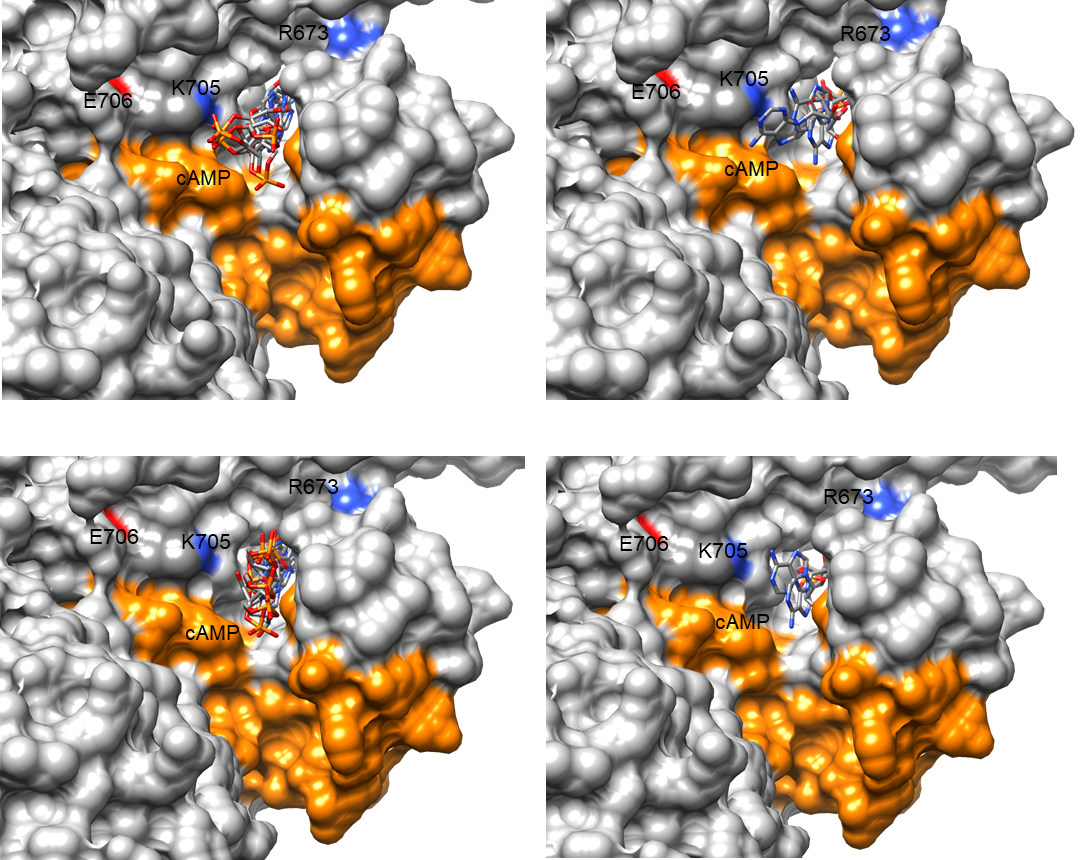
**

**Supplementary Figure S3**

| **AtLARP1A cAMP docking solutions** | | | | | | | |
| --- | --- | --- | --- | --- | --- | --- | --- |
| Mode  1^st^ run | Affinity | Distance from best mode | | Mode  2^nd^ run | Affinity | Distance from best mode | |
|  | (kcal/mol) | rmsd l.b. | rmsd u.b. |  | (kcal/mol) | rmsd l.b. | rmsd u.b. |
| 1 | -5.1 | 0.000 | 0.000 | 1 | -5.1 | 0.000 | 0.000 |
| 2 | -4.7 | 1.570 | 2.399 | 2 | -4.6 | 2.245 | 2.339 |
| 3 | -4.5 | 2.357 | 3.889 | 3 | -4.6 | 1.969 | 3.184 |
| 4 | -4.5 | 2.076 | 3.465 | 4 | -4.3 | 4.521 | 6.315 |
| 5 | -4.5 | 2.013 | 2.582 | 5 | -4.1 | 1.785 | 2.540 |
| Ave ± SD | -4.66 ± 0.26 | | | Ave ± SD | -4.54 ± 0.38 | | |
| **Mean ± SEM** | **-****4.60 ± 0.06** | | | | | | |

Only solutions with “correct binding pose” are shown and considered in free energy calculations

| **AtKING1 cAMP docking solutions** | | | | | | | |
| --- | --- | --- | --- | --- | --- | --- | --- |
| Mode  1^st^ run | Affinity | Distance from best mode | | Mode  2^nd^ run | Affinity | Distance from best mode | |
|  | (kcal/mol) | rmsd l.b. | rmsd u.b. |  | (kcal/mol) | rmsd l.b. | rmsd u.b. |
| 1 | -4.8 | 4.997 | 7.834 | 1 | -5.9 | 0.000 | 0.000 |
| 2 | -4.8 | 4.135 | 6.199 | 2 | -5.7 | 1.884 | 2.191 |
|  |  |  |  | 3 | -5.7 | 3.654 | 4.575 |
|  |  |  |  | 4 | -5.4 | 1.875 | 2.495 |
|  |  |  |  | 5 | -5.3 | 2.696 | 3.657 |
|  |  |  |  | 6 | -5.2 | 2.841 | 4.185 |
|  |  |  |  | 7 | -5.2 | 1.987 | 2.233 |
| Ave ± SD | -4.80 ± 0.00 | | | Ave ± SD | -5.49 ± 0.28 | | |
| **Mean ± SEM** | **-5.14 ± 0.34** | | | | | | |

Only solutions with “correct binding pose” are shown and considered in free energy calculations

**AtLARP1A AtKING1**

**
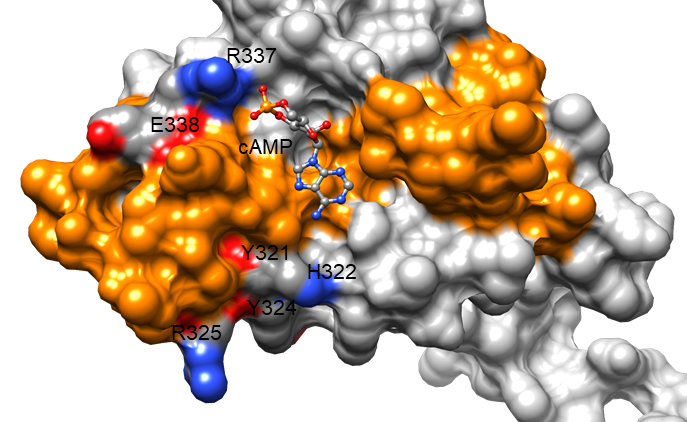

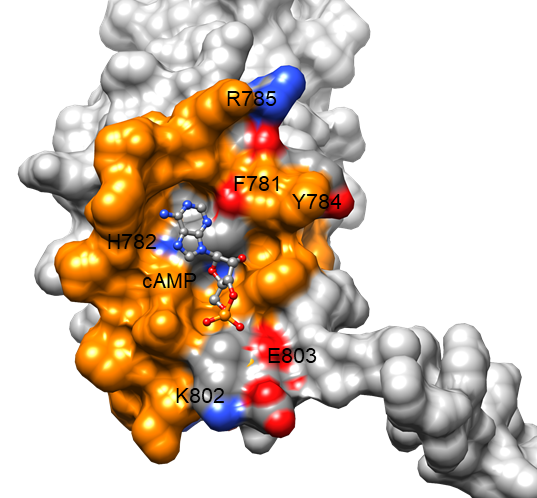
**

AtLARP1A *Arabidopsis thaliana* LA RNA-binding protein (TAIR ID: At5g21160; UniProt ID: Q940X9)

AtKING1 *Arabidopsis thaliana* SNF1-related protein kinase regulatory subunit gamma 1 (TAIR ID: At3g48530; UniProt ID: Q8LBB2)

**Positive clusters, docking data and representative images of** **AtLARP1A and AtKING1 PDE centers docked with cAMP.**

At the predicted PDE regions, AtLARP1A shares 57.5% identity to a LARP1-unique domain DM15 crystal structure from *Homo sapiens* (PDB ID: 4ZC4) and AtKING1 shares 29.0% identity to a Bateman2 domain Snf4 from *Saccharomyces cerevisiae* (PDB ID: 2NYC). The PDE regions of AtLARP1A and AtKING1 were generated by homology modeling against the aforementioned crystal structures using the MODELLER (ver. 9.25) software [3]. AtLARP1A and AtKING1 were docked with cAMP at their PDE centers. A total of 18 solutions across two independent simulations generated by AutoDock Vina (ver. 1.1.2) [4] were evaluated in terms of free energies and binding poses. Orientations and binding poses were analyzed with the UCSF Chimera (ver. 1.10.1) [5]. Chimera is developed by the Resource for Biocomputing, Visualization, and Informatics at the University of California, San Francisco (supported by NIGMS P41-GM103311). In docking simulations, all bonds in the cAMP ligand were allowed to move freely but the protein was set rigid. Docking orientations of cAMP were evaluated based on a pre-determined "correct binding pose" described in the legend of Supplementary Figure S2. For AtLARP1A and AtKING1 cAMP docking, only positive binding poses were shown and from which, the mean binding affinities were determined to be -4.60 ± 0.06 for AtLARP1A and -5.14 ± 0.34 kcal/mol for AtKING1, respectively.

**Supplementary Table S1**

**List of *Arabidopsis thaliana* candidate PDEs**

Search pattern [YFW] H x [YFW] R x {20,40} [HRK] [DE]

Dataset searched TAIR10 Proteins (protein)

| **Gene ID** | **Region** | **Hit pattern** | **Description** |
| --- | --- | --- | --- |
| At1g17330.1 | 27-64 | WHVWRVRDLALSIAREEGLSSNSDSMEIVELAALLHD | Metal-dependent phosphohydrolase |
| At1g51160.1 | 93-127 | FHSFRTNTYKLSFMETPSGIKIILVTHPKTGDLRE | TRAPP protein BET5 homolog |
| At1g77270.1 | 638-674 | YHRYRKDLPRALNLLRGFFFYLENIVLEGAFKPWLHE | Hypothetical protein |
| At1g78240.1 | 116-145 | YHNYRRLQEQLVSDLWDIGEISLGPNRWKE | TSD2 (TUMOROUS SHOOT DEVELOPMENT2) |
| At2g04940.1 | 269-302 | WHLWRRIYDLYLGNNQFAVVENPGFWNWTFTVKD | Phospholipid scramblase |
| At2g15270.1 | 82-117 | FHQYRQMRRREQDRLTRMDIDYNKRLKMAEFTIRRE | PRKR-interacting protein |
| At2g18020.1 | 186-228 | YHKYRVKRNSWPKVRGVAMNPVEHPHGGGNHQHIGHASTVRRD | EMB2296, EMBRYO DEFECTIVE 2296 |
| At2g24740.1 | 634-670 | YHSFRWNYEPELLCEDACEQVSEDANLPTQVLISAKE | SDG21, SET DOMAIN GROUP 21, SU(VAR)3-9 HOMOLOG 8 |
| At2g26710.1 | 88-119 | YHHWRKIYGATFLVWFGPTFRLTVADPDLIRE | BAS1, CYP72B1, CYP734A1, PHYB ACTIVATION TAGGED SUPPRESSOR 1 |
| At3g17550.1 | 226-263 | YHFFRVNGPEESNSYTEEKRDESKNNGGLANVLKLLKE | Haloacid dehalogenase-like hydrolase (HAD) superfamily protein; |
| At3g21140.1 | 262-289 | FHYFRMQNISDIYFIGGFGTVAWVDVKE | Pyridoxamine 5-phosphate oxidase family protein |
| At3g44080.1 | 63-97 | YHRYRPDIQKSFEDFVERTLAFRGHIKTFSLKSRE | F-box family protein |
| At3g48530.1 | 321-365 | YHDYRSITTKNFLVSVREHLEKCGDTSAPIMSGVIACTKNHTLKE | KINγ, KING1, SNF1-RELATED PROT. KINASE REG. SUBUNIT GAMMA 1 |
| At3g50140.1 | 152-178 | YHKWRAVNMVMKRTKQGIEMYIDAMKE | Transmembrane protein, putative (DUF247) |
| At3g50190.1 | 135-161 | YHKWRAVNMVMKRTKQGIEMYIDAMKE | Transmembrane protein, putative (DUF247) |
| At3g56060.1 | 3-36 | FHIFRFFQFILVAVFIFHDSCYSDEAGNYRFMKD | Glucose-methanol-choline (GMC) oxidoreductase family protein |
| At3g57200.1 | 334-367 | WHNYRKSPNEVKLEEAAVLHYTYPRFSDLTSRRD | Glycosyltransferase-like protein |
| At4g17670.1 | 59-92 | FHDFRFDNSYYGYGQPHFLDSCFLCKKRLGDNRD | Senescence-associated family protein (DUF581) |
| At4g20760.1 | 208-234 | WHSYRASKSALNQLTKNVSVELGRRKD | NAD(P)-binding Rossmann-fold superfamily protein |
| At4g24880.1 | 225-256 | YHKFRFSVVPFYNCDQSGLHSAYTGSLPYVRD | Snurportin-1 protein |
| **At4g33530.1** | **667-706** | **YHLFRCVARYGYKDVRKESHQAFEQILIESLEKFIRKE** | **Potassium transporter - KUP5** |
| At4g36130.1 | 186-228 | YHKYRVKRNCWPKVRGVAMNPVEHPHGGGNHQHIGHASTVRRD | Ribosomal protein L2 family |
| At4g39560.1 | 153-186 | YHTWRDAPNMLVERNSHAANVIDGKIYVAGGSRD | Galactose oxidase/kelch repeat superfamily protein |
| At5g09400.1 | 668-705 | YHLFRCIARYGYKDARKETHQAFEQLLIESLEKFIRRE | ATKUP7, K+ UPTAKE PERMEASE 7, KUP7 |
| At5g12080.1 | 436-462 | YHVFRNVAQPFFNYIEEEDLLRFMIKE | ATMSL10, MECHANOSENS. CHANNEL OF SMALL CONDUCTANCE |
| At5g18520.1 | 407-435 | FHMFRPEEKNEYFAVDDDEEEAAALALRD | CAND7, CANDIDATE G-PROTEIN COUPLED RECEPTOR 7 |
| At5g21160.1 | 781-815 | FHHYRGKEEPITKHPELEKLLKEEFRSIDDFRAKE | ATLARP1A, LA RELATED PROTEIN 1A, LARP1A |
| At5g36930.1 | 355-399 | WHAFRTSEPPKEFLQHSEEVVTYCAGLPLAVEVLGAFLIERSIRE | Disease resistance protein (TIR-NBS-LRR class) family |
| At5g47060.1 | 85-113 | FHDFRFDIQQPHFLDSCFLCKKPLGDNRD | Hypothetical protein (DUF581) |
| At5g52460.1 | 295-338 | WHGYRGNEEETQLIRYIFANAKCLKKVTVTSIWEHFQSSILKRE | FBD, F-box and Leucine Rich Repeat domains containing |
| At5g52460.2 | 227-254 | WHGYRGNEEETQLIRYIFANAKCLKKRE | FBD, F-box and Leucine Rich Repeat domains containing protein |
| At5g53620.1 | 588-620 | WHDYRKKYGKLDDFVASHLELFSIEDDYIQVRD | RNA polymerase II degradation factor |

**Bolded** PDE candidate AtKUP5 is experimentally tested and underlined candidates KUP5, KUP7, LARP1A and KING1 are computationally assessed in this study.

**Supplementary Methods**

**The making of the PDE catalytic center search motif**

At the starting point are the 3 annotated plant PDEs, two from lower plants (*Physcomitrella patens* and *Marchantia polymorpha*) and the experimentally confirmed PDE from *Arabidopsis thaliana* (see below).

We have identified the catalytic center (yellow) which contains 3'5'-cyclic nucleotide phosphodiesterase catalytic domain (pfam00233) in *Physcomitrella patens* and marked the residues with essential catalytic functions in red.

>Physcomitrella_patens:112285817 K13293 cAMP-specific phosphodiesterase 4 [EC:3.1.4.53] uncharacterized protein LOC112285817 isoform X1- contains pfam00233 (evalue = 2.30e^-90^, 3'5'-cyclic nucleotide phosphodiesterase – the protein also harbors a c-terminal adenylate cyclase, class 3 domain cog2114)

MSEQENSAPKSGTFQVSEGEHKSLQGIESWSFDVFQVEDDQLPMLVEKIFRSLNIFNNFPIDMHKFRAFVRAMVARYQPNP**YH**C**FR**HACDVLHAVYLILTLAEGAQRLNQMEIFAVALAAVC**HD**VDHPGLTNAFLVAASDPISLRYNDKAVLESHHAATAFLTMRGHDNVNIMFSLDEPHQRHLRRLVLSCILATDMGVHAEIISSFTGRLNDRRPFELSAPYTPPGPMPSKAPEPSDTGDTKTLRADEASMSSSAAPPNDGNTNQPPPELKSPNPKYLLPPLTSSSDVMLYMQMIIKCADLSNVVKPFFLSKRWSALLLLEWFRQGDIERELGMRVSKFMERSEPTTLMDMTIGCIDFVAKPMFEATAVLLPHLHDEALANLTLNRSLWSSFSMGGRRGSEVARNILGPFLPPPITIDGIAEKFGEAAVKMDISSSSLHYDMYLPGVEKESLPMAPLQLLPTLSPEEPTPLINELRMDEHHPSLLALDSSGDKSQSINKGPLRQDRESSILGGAAPLSLLRVNRNASLDASSGDGNNLALRSFVALRRNTVDERNRKPSSISLPPPGYKGTEMMTDPSKNVALDIQSLAFIQDSAWDNRKFWDSIRRHPNGKQLHAFLESRLWISINIIATFVAMFANDFTKAILPKAADAYETHILSICLFIFFLEIIMLSILSQGYFLGFFFWLDLIGSVSLLTVITNVYTQNLVIARTGRAVKAMNRLSKSMQATPLSSMFPITYILKVFHYKNVVVDDDEDEYFHVVSSTKPSQVWTELSELTAQKLIIGLLVMMIVSPLFRDNHRDLGPKMSLNTLDEFHGCTAHFNETIDRIQTFNMLHGYNLLYMGINGMCCGVSCALDSATNFMQIVPSKGADGKKEAQLEYRTVELKSVLSDSNRTEALYSIRSELRTKHALNMAMTVLWLIILGAWSFVLSNDSNRLLIQPIERMVEIVKELSDDPVSFAARANVKQPAISSVGQVMETRVVEAALVKIASLSKVALGDAGMDILSVNLKGGSEFNPMIPGKKIRACFGFCDIRNFTDATECLQEDVMMFVNKIADVVHNKVVFHSGFPNKNIGDAFLLVWKKSAADNIQKTRGTNSFADRALQSFLDIIQCIETSQTLAEYAMHPAIQKRMPGYKIHMGFGLHVGWAIEGAIGSAHKVDPTYLSPHVNVSSRLEAATKQYGVMMLISETVVAELTKSSLRHCCRKLDRITVKGSAAPIIIYTFDLPLFQQDLKGNPADYRALFENAVDNYIEGNWSSALEKLEECLQLWPTDKPGHVLLNFMASHNYRIPASWKGYRELTEK

The motif is made by the inclusion of chemically related amino acids in the key positions and the relaxing of the gaps between the key amino acids:

[**YFW**]-**H**-x-[**FWY**]-**R**-x(20,40)-[**HRK**]-[**DE**]

This motif is present in the other two annotated PDEs:

>Marchantia polymorpha: [PTQ35772.1](https://www.ncbi.nlm.nih.gov/protein/PTQ35772.1?report=genbank&log$=protalign&blast_rank=1&RID=XEVY6Z5U01R), PDE_domain_1 - contains pfam00233 evalue = 2.30e^-90^, 3'5'-cyclic nucleotide phosphodiesterase

WTFDIFQLDDDSLPKMVEKIFRELGLFDNFPLDVKKVRAFTNAMVMRYQPNP**YH**N**FR**HACDVLHAVYLILTLVDGRKKLSHLEVFALALAALC**HD**VDHPGLTNAFLVATYDPLALRYNDRAVLESHHAATCFITMRGNDSLNLLAGLSEEEQRHMRKLMIVLILATDMGEHARILREVGERVQDLRPFEQSPFYTPPGCLSPILRDAESSSSGNTTAGAKSSDAPPSPKRLPDKVYKNPLSPSPPIQSTSDVMLLIQLIIKCADISNVVKPFFLSKRWAALLLLEWFRQGEIEKQLGLPISKFMDREDPSTLMAMTCGCIDYIAKPMYEVMTKLLPRMHENVLVNLN

>At1g17330_PDE – contains cog1418 evalue = 6.17e^-24^, HD superfamily phosphodieaserase, includes HD domain of RNase Y

MAAKTMRKAEELVEKAMKGNDASHDA**WH**V**WR**VRDLALSIAREEGLSSNSDSMEIVELAALL**HD**IGDYKYIRDPSEEKLVENFLDDEGIEETKKTKILTIINGMGFKDELAGVALCESLPEFGVVQDADRLDAIGAIGIARCFTFGGSRNRVLHDPEIKPRTELTKEQYIKREEQTTINHFHEKLLKLKKLMKTEAGKRRAEKRHKFMEEYLKEFYEEWDGST

Note: The above strict motif is not found in the annotated candidate PDE from poplar (see below) and, this may make it a case for future extensions of the motif. The putative catalytic residues are marked, and a possible extension of the motif is proposed below.

>*Populus euphratica*: XP_011015101.1 - contains pfam00233 evalue = 1.70e^-96^ predicted high affinity cAMP-specific and IBMX-insensitive 3',5'-cyclic phosphodiesterase 8A-like [Populus euphratica](553 AA)

MLTNSTPALAFITFTITTRTSLPRSTSHIVLVCDPGSLNEVGGARGSLHSIRRGSLDVRSVASDGLRRTSLAKLQGLPLEAPITKVISLLSAASEGAPPSTVACIDRAVDMLRTSELYSPQMRDEPKLRMEDPIATDLIGALLSQGPTNVLSSRRSSNDSTVVRSQTNRSNIKHKVSSFQTSGQLRELLDTSLSWDFEIFRLEDLTRGRPLAHLGLTLMGGRFDVCASLECDERTLVNWLTVIEMHYHAVNTYHNSTHAADVLQAVSYFLEKDRLKTLLEPLEAAAALISAAAHDVDHPGFSSAFLCNSRHPLALLYNDFSVLESHHAAITFKLTLNDDRANIFKNLDRDTYKTVRHNVIDMILATEMTRHFEHLAKFVNAFDTKTQGSKEDMHSDSTALSLPENVVLIKRMMIKCADVSNASRPQKFAFEWARRIAEEYFLQTDEEKAKDLPVVMPMFDRASCSIPRSQIGFIDFIIMDMMEAWAAFIEMPELVTHARNNHQRWKELDEVGVTTIADVKRAQRQHAIQAAPTSTSQPTAEE

Extended motif:

[YFW]-H-x-[FWY**S**]-x(0,1)-[HRK]-x(20,40)-[HRK]-[DE]

>AtKUP5_may_contain a PDE catalytic center motif -[YFW]-H-x-[YFW]-R-x(20,40)-[HRK]-[DE]:

MFHVEEESSGGDGSEIDEEFGGDDSTTSLSRWVFDEKDDYEVNEDYDDDGYDEHNHPEMDSDEEDDNVEQRLIRTSPAVDSFDVDALEIPGTQKNEIEDTGIGKKLILALQTLGVVFGDIGTSPLYTFTVMFRRSPINDKEDIIGALSLVIYTLILIPLVKYVHFVLWANDDGEGGTFALYSLICRHANVSLIPNQLPSDARISGFGLKVPSPELERSLIIKERLEASMALKKLLLILVLAGTAMVIADAVVTPAMSVMSAIGGLKVGVGVIEQDQVVVISVSFLVILFSVQKYGTSKLGLVLGPALLLWFFCLAGIGIYNLVKYDSSVFKAFNPAYIYFFFKRNSVNAWYALGGCVLCATGSEAMFADLSYFSVHSIQLTFILLVLPCLLLGYLGQAAYLSENFSAAGDAFFSSVPSSLFWPVFLISNVAALIASRAMTTATFTCIKQSIALGCFPRLKIIHTSKKFIGQIYIPVLNWSLLVVCLIVVCSTSNIFAIGNAYGIAELGIMMTTTILVTLIMLLIWQTNIIVVSMFAIVSLIVELVFFSSVCSSVADGSWIILVFATIMFLIMFVWNYGSKLKYETEVQKKLPMDLLRELGSNLGTIRAPGIGLLYNELAKGVPAIFGHFLTTLPAIHSMVIFVCIKYVPVPSVPQTERFLFRRVCPRS**YH**L**FR**CVARYGYKDVRKESHQAFEQILIESLEKFIR**KE**AQERALESDGDHNDTDSEDDTTLSRVLIAPNGSVYSLGVPLLAEHMNSSNKRPMERRKASIDFGAGPSSALDVEQSLEKELSFIHKAKESGVVYLLGHGDIRATKDSWFLKKLVINYLYAFLRKNSRRGITNLSVPHTHLMQVGMTYMV

In addition, other A.t. proteins also contain the motif and may be candidate PDEs:

At1g78240 Pectin methyltransferase QUA2

At2g18020 Ribosomal protein

At3g48530 SNF1-related protein kinase regulatory subunit gamma-1

At3g57200 Glycosyltransferase-like

At5g09400 AtKUP7

At5g12080 Mechanosensitive ion channel protein 10

At5g21160 LA-related protein 1A, LARP1a

If we allow and additional gap between the last 2 amino acids (in blue), two other KUPs can be included in the PDE candidate list:

[YFW]-H-x-[YFW]-R-x(20,40)-[HRK]-x(0,1)-[DE]

AtKUP5 YHLFRCVARYGYKDVRKESHQAFEQILIESLEKFIRKE

AtKUP7 YHLFRCIARYGYKDARKETHQAFEQLLIESLEKFIRRE

AtKUP10 FHMFRCVARYGYRDLHKKDDD-FEKRLFESLFLFLRLE

AtKUP11 FHMFRCVARYGYRDLHKKDDD-FEKRLFESLFLYVRLE

:*:***:*****:* :*: .: **: *:*** ::* *

Incidentally, this motif occurs 839 times in 829 sequences in Prosite. Of these hits, 97 are annotated PDEs.

**Expression vector construct**

Total RNA was isolated from leaves and stalk of *A. thaliana* using RNeasy Plant Mini Kit (Qiagen). The first-strand cDNA for RT-PCR was synthesized using GoScript™ Reverse Transcription System (Promega) following the manufacturer’s instructions. In order to construct the expression plasmids for the GST-AtKUP5^573-855^, a cDNA fragment encoding the PDE domain together with calmodulin binding site (573-855) was amplified by RT-PCR using the specific primers:

- *AtKUP5^573-855^* (forward)

5ʹ-GGATCCCCAGGAATTCCCATGTTTGTTTGGAACTACGGGAG-3ʹ,

- *AtKUP5^573-855^* (reverse)

5ʹ-GATGCGGCCGCTCGAGAATCATACCATATAAGTCATTCCAACT-3ʹ,

The PCR reactions were performed with a cDNA as a template, forward and reverse primers and CloneAmp HiFi PCR Premix (Takara). The amplified DNA fragment (855 bp) was purified using Agarose-Out DNA Purification Kit (Eurx). The amplified PCR product was cloned into pGEX-6P-2 vector (GE Healthcare) in the *Eco*RI – *Xho*I restriction sites using an In-fusion Cloning kit (Takara). Both, GST-AtKUP5(K705A) and GST-AtKUP5(K705D), mutants were constructed by site directed mutagenesis using QuikChange II XL Site-Directed Mutagenesis Kit (Agilent). Specific primers used in the reaction were:

- *AtKUP5^K705A^* (forward)

5ʹ-CACGCTCCTGTGCTTCCGCACGTATAAATTTTTCTAGACTCT-3ʹ

- *AtKUP5^K705A^* (reverse)

5ʹ-TGAGAGTCTAGAAAAATTTATACGTGCGGAAGCACAGGAGCGTG-3ʹ

- *AtKUP5^K705D^* (forward)

5ʹ-GTGCACGCTCCTGTGCTTCATCACGTATAAATTTTTCTAGACTCT-3ʹ

- *AtKUP5 ^K705D^* (reverse)

5ʹ- AGAGTCTAGAAAAATTTATACGTGATGAAGCACAGGAGCGTGCAC-3ʹ

**Expression and purification of the recombinant protein**

The resulting plasmid was introduced into the *E. coli* BL21(DE3) pLysS competent cells (Promega) in order to produce the fusion protein with glutathione-S-transferase (GST) affinity tag. The transformants were grown in LB medium (500 ml) containing ampicillin (100 μg/ml) and 2 % glucose at 37 °C. Fusion protein expression was induced by adding isopropyl-ß-D-thiogalactopyranoside (IPTG) to a final concentration of 0.5 mM at OD_600_ = 0.6 and incubating the culture at 24 °C for 3.5 h. The bacteria were harvested by centrifugation and the pellet was suspended in lysis buffer (50 mM Tris-HCl pH 8.0, 150 mM NaCl, 5 mM EDTA, 5 mM EGTA, 1 % (v/v) Triton X-100, 1 mM PMSF, 0.2 mg/ml lysozyme) and disrupted by sonication. The cell extract was centrifuged at 12,000 × g for 35 minutes and the supernatant was loaded onto a glutathione-Sepharose 4B beads (GE Healthcare). Afterwards the column was washed multiple times with buffer containing 50 mM Tris-HCl (pH 8.0), 150 mM NaCl and the GST fusion protein was eluted with 10 mM glutathione in 50 mM Tris-HCl (pH 8.0). The recombinant calmodulins (CaM1, 9) were purified according to [17]. The recombinant CaM2 was purchased from Sigma. The homogeneity and purity of eluted protein fraction was analyzed by SDS–PAGE electrophoresis (10 % gel) with the Coomassie Blue gel staining [6].

**Surface Plasmon Resonance analysis**

Surface plasmon resonance experiment was performed at 25°C on Reichert SR7500DC dual channel surface plasmon resonance (SPR) apparatus. Bare gold sensor discs were used. The flow rate of buffer and protein solution throughout the analysis was 10 µl/min. The GST-tag which permit oriented immobilization of GST-tagged proteins on the gold discs (thickness 0.9 mm) were used. GST-AtKUP5^573-855^ immobilization on gold discs was carried out as follows: the gold discs were put in in an ethanolic solution containing glutathione (10 μM) and β-mercaptoethanol (1 mM) for 3h at room temperature. The coated discs were then washed with ethanol, water and dried. Such prepared gold disc was mounted into SPR instrument. For attaining steady baseline in sensogram the disc surface was washed by 30 mM phosphate buffer for 20 minutes. For immobilization of GST-AtKUP5^573-855^, its solution was injected (0,625 µM) for 20 minutes. After that, solution of CaM9 (1 µM) was injected to gold disc surface (10 minutes) for checking the interaction with phosphodiesterase. In the next step, solution of CaM9 (1 µM) with 1 µM Ca^2+^ ion was injected for 10 minutes. Both proteins were prepared in 30 mM phosphate buffer. Between the injections of proteins, the surface of gold disc was washed with 30 mM phosphate buffer solution.

**Supplementary References**

[1] Al-Younis I, Wong A, Lemtiri-Chlieh F, Schmöckel S, Tester M, Gehring C, et al. The *Arabidopsis thaliana* K^+^-uptake permease 5 (AtKUP5) contains a functional cytosolic adenylate cyclase essential for K^+^ transport. Front Plant Sci 2018;9:1645.

[2] Al-Younis I, Wong A, Gehring C. The *Arabidopsis thaliana* K+-uptake permease 7 (AtKUP7) contains a functional cytosolic adenylate cyclase catalytic centre. FEBS Lett 2015;589(24 Pt B):3848-3852.

[3] Šali A, Blundell TL. Comparative protein modelling by satisfaction of spatial restraints. J Mol Biol 1993;234(3):779-815.

[4] Trot O, Olson AJ. Software News and Update AutoDock Vina: Improving the Speed and Accuracy of Docking with a New Scoring Function, Efficient Optimization, and Multithreading. J Comput Chem 2010;31(2):455-461.

[5] Pettersen EF, Goddard TD, Huang CC, Couch GS, Greenblatt DM, Meng EC, et al. UCSF Chimera - A visualization system for exploratory research and analysis. J Comput Chem 2004;25(13):1605-1612.

[6] Świeżawska B, Duszyn M, Kwiatkowski M, Jaworski K, Pawełek A, Szmidt-Jaworska A. *Brachypodium distachyon* triphosphate tunnel metalloenzyme 3 is both a triphosphatase and an adenylyl cyclase upregulated by mechanical wounding. FEBS Lett 2020;594(6):1101-1111.
